# Combined Aspiration and Stent Retriever Thrombectomy for Distal Carotid Artery Occlusion Using Balloon Guide versus Non-Balloon Guide Catheter

**DOI:** 10.3390/jcm13071978

**Published:** 2024-03-29

**Authors:** Ender Uysal, Bade von Bodelschwingh, Omer Naci Tabakci, Celal Ilker Basarir, Serpil Bulut

**Affiliations:** 1 Radiology Clinic Antalya, Antalya Training and Research Hospital, University of Health Sciences, Antalya 07100, Turkey; 2 Radiology Clinic, Istanbul Sisli Hamidiye Etfal Training and Research Hospital, University of Health Sciences, Istanbul 34098, Turkey; badevonbodelschwingh@gmail.com (B.v.B.); omertabakci@gmail.com (O.N.T.); 3 Neurology Clinic Istanbul, Istanbul Sisli Hamidiye Etfal Training and Research Hospital, University of Health Sciences, Istanbul 34098, Turkey; drilkerbasarir@gmail.com (C.I.B.); serpilbulut@yahoo.com (S.B.)

**Keywords:** acute ischemic stroke treatment, thrombectomy, balloon guide catheter, non-balloon guide catheter, combined treatment

## Abstract

**Background**: The introduction of endovascular thrombectomy dramatically changed acute stroke management and became the standard treatment. Balloon guide catheters provide flow arrest during the clot retrieval process and have several advantages.This study aimed to compare balloon guide catheters (BGCs) versus non-balloon guide catheters (NBGCs) as a part of a combined treatment modality in patients presenting with acute ischemic stroke. **Methods**: This retrospective study included *n* = 65 patients who underwent a combined endovascular stroke treatment for distal internal carotid artery (ICA) occlusion. Patients underwent aspiration and stent retriever thrombectomy with the use of BGCs (Group 1, *n* = 27) or NBGCs (Group 2, *n* = 38). **Results**: The groups were compared for outcomes: the National Institutes of Health Stroke Scale (NIHSSS) score change, successful recanalization, good functional outcome at three months, and in-hospital mortality. **Conclusion**: The two groups didn’t differ in terms of the NIHSS score change compared to baseline (*p* > 0.05). Moreover, there were no significant differences between the two groups in terms of the successful recanalization rate, three-month favorable functional outcome rate, and in-hospital mortality (*p* = 0.292, *p* = 0.952, *p* = 0.178), respectively. Further prospective studies with a larger number of patients and better methodology are warranted.

## 1. Introduction

Stroke is a leading cause of death in developed countries, ranking the fifth in the United States [1]. Acute internal carotid artery occlusion accounts for 4–15% of all ischemic strokes [2]. Distal internal carotid artery (ICA) occlusions presenting with acute stroke have devastating clinical outcomes with high mortality rates [3,4]. It has been known for two decades that intravenous thrombolysis is an effective treatment option for all patients with acute ischemic stroke. However, intravenous thrombolysis is not a treatment option for all patients due to the length of time between the onset of symptoms and presentation to the healthcare system and contraindications [2]. In addition, a study has shown that the time it takes to reach a health center is not suitable for IV thrombolysis in the majority of stroke patients [5].

The introduction of endovascular thrombectomy dramatically changed acute stroke management and became the standard treatment for large vessel occlusions [6,7,8]. Mechanical thrombectomy with or without thrombolytic agents is the first choice of treatment since the prognosis of patients solely receiving thrombolytics is not favorable [9]. Thrombectomy procedures in combination with local aspiration catheters, stent retrievers, or both have previously been described [10,11,12,13]. Recent studies showed that combining continuous aspiration and mechanical thrombectomy with stent retrievers resulted in increased recanalization rates and better clinical outcomes [13].

Balloon guide catheters provide flow arrest during the clot retrieval process, which may have potential beneficial effects such as the low risk of clot fragmentation and distal emboli, as well as more effective revascularization [14,15]. The Systematic Evaluation of Patients Treated with Neurothrombectomy Devices for Acute Ischemic Stroke (STRATIS) results showed that use of the balloon guide catheter (BGC) was an independent predictor of the first-pass effect (FPE) and functional independence [16,17]. In addition, several meta-analyses demonstrated superior technical and clinical outcomes with balloon guide catheters when compared to non-balloon catheters [18,19,20,21]. However, the beneficial effects of the use of balloon guide catheters when they are exclusively used as a part of a combined treatment modality remain to be elucidated. Furthermore, anterior circulation occlusions may differ according to the specific site of occlusion, so the anatomical differences and clot burden between the middle cerebral artery (MCA) and intracranial internal carotid artery occlusions may require them to be treated with different thrombectomy techniques. There have been studies that looked at intracranial ICA occlusions, often limited by their relatively small sample [10,22] and lack of subanalysis focusing on the occlusion site [16].

The superiority of different therapeutic approaches employed in mechanical thrombectomy regarding patient outcomes remains to be fully elucidated. This study aimed to evaluate a combined endovascular treatment in patients with distal ICA occlusion and to compare the use of a BGC and a non-balloon guide catheter (NBGC) as a component of treatment in terms of both recanalization success and clinical outcomes.

## 2. Methods

In this retrospective study, data of all patients (*n* = 200) who underwent endovascular stroke treatment at a single private stroke center between April 2018 and October 2020 were analyzed, and patients with anterior circulation acute ischemic stroke caused by distal ICA occlusion (T or L type) requiring mechanical thrombectomy (*n* = 65) were included. T or L occlusion type is a classification according to the shape of the clot in the occluded vessel [23]. Diagnosis was made using either computerized tomography or magnetic resonance imaging. In patients admitted within 6 h after the onset of symptoms, the decision for mechanical thrombectomy was made using initial computerized tomography imaging. In patients who presented with a wake-up stroke or in those admitted beyond 6 h after the onset of symptoms, the decision to proceed with mechanical thrombectomy was made after the evaluation of an initial magnetic resonance imaging (MRI) using a 1.5 Tesla MAGNETOM Avanto scanner (Siemens, Erlangen, Germany). Patients admitted within 4.5 h following the onset of symptoms were treated with a standard dose of tissue plasminogen activator (tPA) (0.9 mg/kg) if there were no contraindications according to European guidelines [24]. For analyses, patients were assigned into one of the two groups based on mechanical thrombectomy method: Group 1, balloon guide catheter group, *n* = 27 (after July 2019); and Group 2, non-balloon guide catheter group, *n* = 38 (before July 2019).

**Figure 1:**
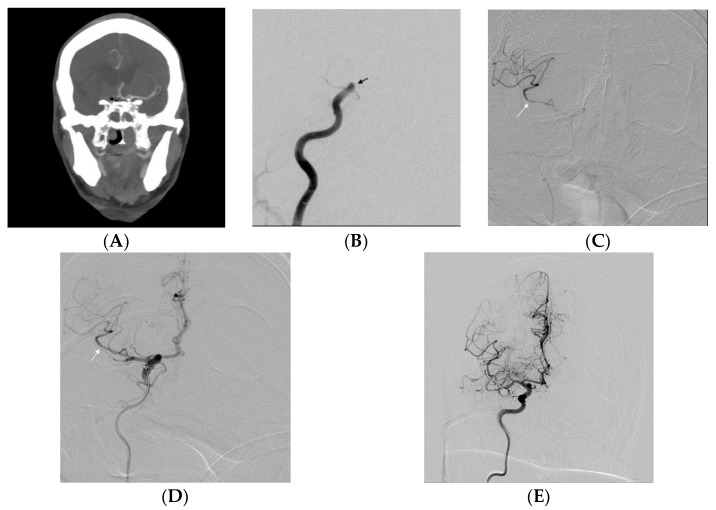
(**A**) Preprocedure CTA of the head (coronal image) indicated that the terminal end of the right ICA was occluded (black arrow). (**B**) DSA of the right common carotid artery revealed that distal ICA was occluded (black arrow), which includes the supraclinoid segment of the ICA. (**C**) After the occlusion site was passed with a microcatheter (white arrow), a gentle contrast injection was performed through the microcatheter to verify the placement beyond the clot. (**D**) A Solitaire 6 × 40 mm stent (white arrow) was used, and 6F intermediate catheter (Sofia) was advanced closer to the thrombus for local aspiration after the stent retriever removal. (**E**) TICI 2B recanalization was observed after a 1-pass.

**Figure 2:**
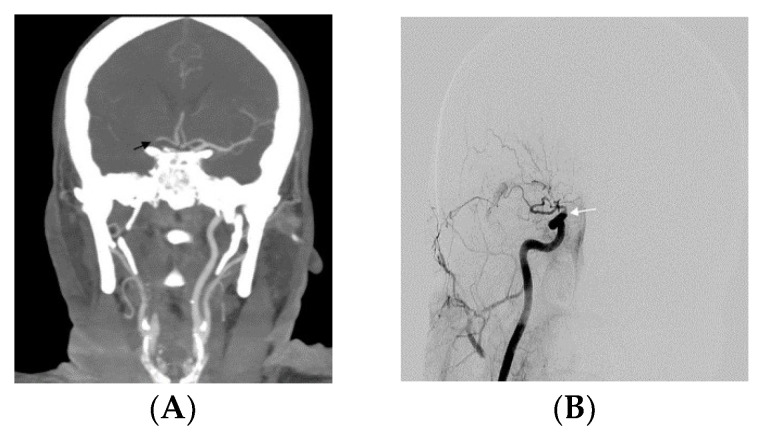

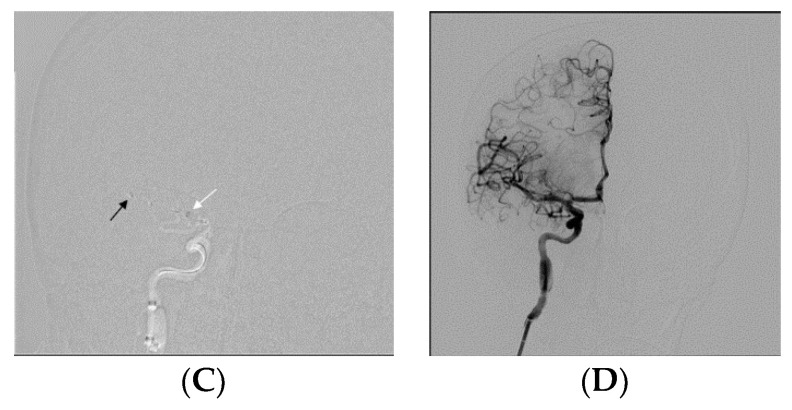
(**A**) Preprocedure CTA of the head indicated that the terminal end of the right ICA was occluded (black arrow) (T occlusion) (**B**) DSA of the right CCA confirmed the distal ICA occlusion (white arrow). (**C**) After the occlusion site was passed with a microcatheter, the stent retriever (black arrow) is completely deployed. 5F Catalyst catheter was connected to a Penumbra pump and was placed beyond the face of the thrombus (white arrow) via roadmap imaging. A balloon guide catheter was inserted into the proximal part of the petrous segment of ICA. (**D**) ICA and MCA were both recanalized with TICI 3 after a 1-pass.

All procedures were performed under general anesthesia by two interventional radiologists using a Siemens Artis Zee biplane system (Erlangen, Germany). For the NBGC group, a 6 French (F) 90 cm Shuttle Sheath (Cook, Bloomington, IN, USA) was advanced to either distal common carotid artery (CCA) or proximal cervical ICA. A Sofia catheter (5F, 6F length 115 cm; Microvention) was used as an intermediate catheter. A marksman microcatheter (eV3, Irvine, CA, USA) was navigated over the Synchro-2 microwire (Stryker, Fremont, CA, USA) or the Traxcess 14 (Microvention, Aliso Viejo, CA, USA) microwire. Solitaire Platinum (sizes 6 × 40 or 4 × 40 mm; Medtronic, Irvine, CA, USA) stent was used as a stent retriever (Figure 1). For the BGC group, an 8F or 9F balloon guide catheter (Cello, Medtronic Neurovascular) was used, and Catalyst (Stryker, Neurovascular) was the intermediate catheter (Figure 2). The same type of microcatheter and microwire was used for both groups. The proximal inner diameter of the Sofia catheter was measured as 0.055 inch and 0.060 inch for Catalyst 6F. However, the difference between the inner diameters of the catheters was negligible. Integration of the procedure with a stent retriever can be expected to reduce the effect of this small variation.

The aspiration catheter, microcatheter, and microwire were advanced intracranially in a triaxial system. After the microcatheter was advanced beyond the site of occlusion, a gentle contrast injection was given through the microcatheter to verify correct placement beyond the clot. After deployment of the stent, the distal aspiration catheter was connected to the aspiration pump and advanced over the stent retriever wire and microcatheter to cover the proximal 1 cm segment of the stent and kept in place for 2–3 min, after which the aspiration catheter and stent were withdrawn through the guide catheter simultaneously. During this maneuver, aspiration from the guide catheter was performed with a 50 mL syringe.

In all patients, neurological status was evaluated using The National Institutes of Health Stroke Scale (NIHSSS) system by a neurologist, prior to treatment and at the time of discharge. Recanalization was evaluated using thrombolysis in cerebral infarction (TICI) scores, where TICI 2b and 3 were defined as successful recanalization. Overall functional status was evaluated using the modified Rankin scale (mRS), and scores between 0 and 2 were considered good functional outcome. In addition, patient demographics, procedure time, tPA status, number of required mechanical thrombectomy attempts, and mortality status were retrieved and recorded.

### Statistical Analysis

Statistical Package for Social Sciences (SPSS, version 27.0) software was used for statistical analysis. Descriptive data are presented in mean ± standard deviation and frequency (percentage). Normality of continuous variables was tested using both hypothesis tests and graphical methods. Intergroup comparisons of continuous variables were carried out using Student’s *t* test for independent samples or Mann–Whitney U test, depending on normality of data. Pearson’s chi-square test or Fisher’s exact test was used for intergroup comparisons of categorical data. To test the intergroup difference for paired observations, Wilcoxon’s signed-rank test was used. Logistic regression was used for multivariate analysis of potential independent predictors of outcomes. A *p* value < 0.05 was considered indication of statistical significance.

## 3. Results

Table 1 shows the demographical and perioperative clinical characteristics of the two study groups. The two groups were similar in terms of the demographical characteristics, NIHSS score at baseline, and intraoperative characteristics. In the current study, the percentage of T occlusion was 66.6% in the BGC group and 68.4% in the NBGC group (*p* = 0.881) In addition, the percentage of L occlusion was 33.3% in the BGC group and 31.5% in the NBGC group (*p* = 0.881). All patients had comorbidity and the most common comorbidity was hypertension (76.9%). None of the patients had a previous major ischemic cerebrovascular event. However, the use of intravenous tPA was more common in the NBGC group (57.9% vs. 25.9%, *p* = 0.011). This discrepancy is due to the fact that the number of patients admitted to the hospital over 4.5 h and underwent mechanical thrombectomy without iv tPA was higher in the BGC group (BGC = 15 (55.5%), NBGC = 12 (31.5%)) (*p* = 0.05). (Table 1) As a consequence of the use of oral anticoagulants, five patients in the BCG cohort and four patients in the NBGC cohort failed to receive iv tPA despite presenting to hospital within the prescribed 4.5 h of symptom onset.

Table 2 shows treatment outcomes by study group. When all patients were considered, a significant decrease was evident in the NIHSS score at discharge, when compared to baseline (11.0 ± 7.3 vs. 16.5 ± 5.5, *p* < 0.001). This significant difference was evident for both the BGC and NBGC groups (*p* < 0.001 for both). However, the two groups did not differ in terms of the NIHSS score change compared to baseline. The two groups were similar in terms of the successful recanalization rate and good functional outcome (mRS 0–2) at three months (*p* > 0.05 for both), (Table 3). No procedure-related mortality was observed; however, 7 (25.9%) patients in the BGC group died before discharge compared to 16 (42.1%) in the NBGC group (*p* = 0.178). Successful recanalization was significantly associated with a lower number of mechanical thrombectomy attempts (2.3 ± 1.2 vs. 3.9 ± 1.3, *p* < 0.001). A multivariate analysis identified the number of mechanical thrombectomy attempts as the only significant independent predictor of successful recanalization (for each unit increase in the number of mechanical thrombectomy attempts OR for successful recanalization: 0.46 (95% CI: 0.25–0.83), *p* = 0.01).

Although good function based on mRS was significantly associated on a univariate analysis with a younger age, lower number of thrombectomy attempts, lower NIHSS score upon admission, and NIHSS improvement at discharge, only a low NIHSS score on admission emerged as the significant independent predictor of good function (for each unit increase in admission NIHSS score OR for good function: 0.84 (95% CI: 0.71–0.99), *p* = 0.03).

On a univariate analysis, older age, the NBGC technique, and unsuccessful TICI were associated with in-hospital mortality. On a multivariate analysis, only a higher age (for each unit increase in age OR for in-hospital mortality: 1.11 (95% CI: 1.01–1.22), *p* = 0.025) and unsuccessful TICI (OR: 16.04 (95% CI: 1.53–167.76), *p* = 0.021) was associated with increased risk of in-hospital mortality.

## 4. Discussion

In this series, we achieved a good recanalization rate (TICI 2b-3) using mechanical thrombectomy combining a stent retriever and aspiration with or without a balloon-guiding catheter in distal ICA occlusions (%88.9 with the BGC vs. %78.9 with the NBGC). There were no significant differences between the cohorts for the outcome measures evaluated, including the rate of successful recanalization, the rate of positive functional outcome at three months, and in-hospital mortality rates.

A number of studies have, so far, compared the use of balloon guide versus non-balloon guide catheters in mechanical thrombectomy after acute stroke. In the study by Valesco et al., the angiographic results and procedure time were improved with the use of balloon guide catheters [25]. They reported recanalization rates of 89.2% and 57.9% for BCG and NBCG, respectively. Only around 17% of their patients had terminal ICA occlusions like the patients in the present study; however, that subgroup also had improved recanalization with the BGC. In that study, a 90-day clinical follow-up is absent, so a comparison with our study findings regarding functional independence is not possible. Moreover, the use of the distal access catheter and double aspiration technique in the BGC and NBGC groups in our study might explain the lack of a significant difference between these two groups, in contrast to the findings of the study by Valesco et al.

In addition to individual studies, four recent meta-analyses compared balloon guide versus non-balloon guide catheters and obtained findings in favor of balloon guide catheter use [18,19,20,21]. In a 2021 meta-analysis with 16 studies and 5507 patients, balloon guide catheters performed better in terms of the first-pass effect, successful reperfusion, good functional outcome, smaller number of passes, procedural time, distal or new emboli, and mortality [21]. Similarly, in another 2021 meta-analysis with 15 studies, better technical and clinical results were obtained with balloon guide catheters, including better revascularization, reduced puncture to revascularization time, fewer endovascular attempts, fewer distal emboli and hemorrhage, a better Rankin scale score at 90 days, and reduced mortality [18]. In the study by Nguyen et al., balloon guide catheters were better in terms of revascularization, good functional outcome at three months, and mortality; however, the two techniques did not differ in terms of reperfusion time, first-pass effect, number of passes, and rescue therapy [20]. In the meta-analysis by Brinjikji et al., balloon guide catheter use was associated with better recanalization and functional outcome, a smaller number of passes, and a shorter procedure time, as well as lower mortality [19]. The above-mentioned studies mostly reported findings, either technical or clinical, in favor of balloon guide catheter use; however, our findings are not in line with them, except for a decreased mortality rate in association with the balloon guide catheter. Nevertheless, it is of note to emphasize that non-balloon guide catheter use did not emerge as a significant independent predictor of mortality on a multivariate analysis, although a univariate analysis showed increased mortality with the non-balloon catheter. An explanation for the lack of difference between the two groups in our study regarding other parameters such as a good functional outcome and recanalization may be the use of a combined approach in both groups (i.e., use of aspiration/intermediate catheter), which may mask the difference, if any. Similarly, in the study by Podlasek et al. [21], the benefits of balloon guide catheters did not persist when there is a combined approach (stent retriever and contact aspiration). Another reason for the lack of difference between groups may be the distribution of ICA and MCA occlusions in the studies. In the present study, all patients had ICA occlusions, which may be another factor that may mask any difference between the groups. This may be particularly important, since a subgroup analysis for the location of the occlusion is lacking in the previous studies.

Several studies have compared different techniques of recanalization for the treatment of intracranial ICA occlusions where direct aspiration, stent retriever, and combined mechanical recanalization were used [10,11,12,13,24,26,27]. Xing et al. compared aspiration to stent retriever without using the balloon guide catheter and showed that the eTICI 2b-3 rate was higher in the aspiration thrombectomy group; however, success rates were not significantly different after using propensity score matching [11]. In another study, McTaggart RA et al. compared a technique named continuous aspiration prior to intracranial vascular embolectomy (CAPTIVE) to the conventional aspiration technique that is very similar to our study [13]. When we compare their results with ours, they found higher rates of TICI2b-3 recanalization (81% traditional vs. 100% CAPTIVE) and increased independence at 90 days (25% traditional vs. 49% CAPTIVE), as compared to our study (TICI2b-3 recanalization, 84% in the BGC group and 78,9% in the NBGC group; and independence at 90 days, 29.6% in the BGC group and 28.9% in the NBGC group) [13]. We can argue that this study included both MCA M1 and ICA occlusions, and the total number of ICA occlusions was only 34, which was lower than our study population (*n* = 65) patients. Furthermore, the mRS results of the ICA occlusion subgroup were not reported separately.

In another study by Maus et al., the efficacy of mechanical thrombectomy using the Tigertriever XL device for terminal ICA occlusions was evaluated in a small group of patients (*n* = 23), and a successful recanalization (TICI 2b-3) rate of 78.3% was obtained [27]. In addition, 26.1% of the patients had a mRS score between 0–2 in the long term. However, patients in that study had a high number of residual occlusions in downstream territories (43.5%), which required the additional use of smaller devices for the further treatment of residual occlusions, prolonging the procedure and increasing the costs.

The most recent study with ICA distal occlusions is by Romano et al., which compared the JET 7 catheter (0.072″) with smaller large-bore catheters for direct aspiration in carotid T occlusions [26]. Successful revascularization (TICI ≥ 2b) was achieved in 94.7% of JET 7 cases and in 75.6% of smaller LBC cases (*p* = 0.148). They reported a good functional outcome (MRS 0–2) at 90 days in 63.2% of the JET 7 cases and in 46.3% of the smaller LBC cases. Supporting our findings, the current study Pero et al. compared the balloon guide catheter with the non-balloon guide catheter using the SAVE (stent-retriever-assisted vacuum-locked extraction) technique in 71 patients with intracranial ICA occlusion (ICA-ICA); the result was that the added benefit of the BGC compared to long sheaths was not remarkable [28].

This study has several limitations. Although we had a good recanalization rate, the functional independence rates at 90 days were relatively low, and this may have been due to patient selection. We included only isolated distal ICA occlusions with a high clot volume in the study; this type of occlusion has the worst prognosis among others [29]. Secondly, the use of the BGC did not alter the clinical outcomes in this study. Finally, the limited sample size due to the low incidence of ICA occlusion in cerebrovascular events and the nonrandomized design are potential limitations.

In conclusion, this study demonstrated that the use of the BGC with combined aspiration and stent retriever thrombectomy in distal ICA occlusions had no significant effect on patient outcomes. Prospective studies with larger numbers of patients and randomization are required to evaluate the possible benefit of the BGC in distal ICA occlusions with a high clot burden.

## Figures and Tables

**Table 1:** Demographical and perioperative clinical characteristics of the patients by study group.

Characteristics	BGC Group *n* = 27	NBGC Group *n* = 38	*p*
Demographics			
Male sex, n (%)	12 (44.4%)	16 (42.1%)	0.851
Age, y	68.7 ± 13.4	68.9 ± 13.4	0.946
Smoking, n (%)	8	12	0.866
Comorbidities			
Hypertension, n (%)	20 (74%)	30 (78.9%)	0.645
Diabetes mellitus, n (%)	10 (37%)	13 (34.2%)	0.814
Hyperlipidemia, n (%)	13 (48.1%)	17 (44.7%)	0.785
Coronary artery disease, n (%)	7 (25.9%)	8 (21%)	0.645
Atrial fibrialation n (%)	5 (18.5%)	4 (10.5%)	0.568
Perioperative parameters			
NIHSS score at baseline	15.4 ± 5.4	17.6 ± 5.2	0.055
tPA use, n (%)	7 (25.9%)	22 (57.9%)	*0.011*
IV tPA ineligible patients n (%)	20 (74%)	16 (42.1%)	*0.010*
Patients who had ≥4.5 h between clinical onset and hospital admission and could not receive tPA, n (%)	15 (55.5%)	12 (31.5%)	0.0532
Patients unable to receive tPA due to contraindications, n (%)	5 (18.5%)	4 (10.5%)	0.568
Number of thrombectomy attempts	2.4 ± 1.4	2.7 ± 1.3	0.224
Time from clinic onset to groin puncture, min	303 ± 120	293 ± 95	0.210
Duration of procedure, min.	65.8 ± 35.0	66.6 ± 27.5	0.637
T occlusion, n (%)	18 (66.6%)	26 (68.4%)	0.881
L occlusion, n (%)	9 (33.3%)	12 (31.5%)	0.881

NBGC, non-balloon guide catheter; BGC, balloon guide catheter; NIHSS, National Institutes of Health Stroke Scale; tPA, tissue plasminogen activator. Unless otherwise stated, data were presented as mean ± standard deviation.

**Table 2:** Treatment outcomes by study group.

Characteristics	BGC Group *n* = 27	NBGC Group *n* = 38	*p*
Change in NIHSS score *	−5.9 ± 5.4	−5.4 ± 6.2	0.647
TICI score, *n* (%)			
0	2 (7.4%)	5 (13.2%)	0.516
1	0 (0.0%)	2 (5.3%)	
2a	1 (3.7%)	1 (2.6%)	
2b	11 (40.7%)	18 (47.4%)	
2c	1 (3.7%)	0 (0.0%)	
3	12 (44.4%)	12 (31.6%)	
Successful recanalization, *n* (%) **	24 (88.9%)	30 (78.9%)	0.292
MRS score	3.6 (2.1%)	4.0 (2.3%)	0.232
Good functional outcome, *n* (%) †	8 (29.6%)	11 (28.9%)	0.952
In-hospital mortality, *n* (%)	7 (25.9%)	16 (42.1%)	0.178

NBGC, non-balloon guide catheter; BGC, balloon guide catheter; NIHSS, National Institutes of Health Stroke Scale; TICI, thrombolysis in cerebral infarction; MRS, modified Rankin scale * Only survived patients with available data were included (*n* = 57). ** TICI score of 2b, 2c, or 3. † MRS score of 0–2 at 90 days. Unless otherwise stated, data were presented as mean ± standard deviation.

**Table 3:** Score on the modified Rankin scale.

	0	1	2	3	4	5	6
BGC (*n* = 38)	5	3	3	2	3	6	16
NBGC (*n* = 27)	4	2	2	3	5	4	7

NBGC, non-balloon guide catheter; BGC, balloon guide catheter.

## Data Availability

All data generated or analyzed during this study are included in this article. Further inquiries can be directed to the corresponding author.

## References

[1] Mendelson S.J., Prabhakaran S. (2021). Diagnosis and Management of Transient Ischemic Attack and Acute Ischemic Stroke: A Review. JAMA.

[2] Mayer L., Grams A., Freyschlag C.F., Gummerer M., Knoflach M. (2020). Management and prognosis of acute extracranial internal carotid artery occlusion. Ann. Transl. Med..

[3] Linfante I., Llinas R.H., Selim M., Chaves C., Kumar S., Parker R.A., Caplan L.R., Schlaug G. (2002). Clinical and vascular outcome in internal carotid artery versus middle cerebral artery occlusions after intravenous tissue plasminogen activator. Stroke.

[4] Christou I., Felberg R.A., Demchuk A.M., Burgin W.S., Malkoff M., Grotta J.C., Alexandrov A.V. (2002). Intravenous tissue plasminogen activator and flow improvement in acute ischemic stroke patients with internal carotid artery occlusion. J. Neuroimaging.

[5] Soto-Cámara R., González-Santos J., González-Berna J., Trejo-Gabriel-Galán J.M. (2020). Factors associated with a rapid call for assistance for patients with ischemic stroke. Emergencias.

[6] Goyal M., Menon B.K., Van Zwam W.H., Dippel D.W., Mitchell P.J., Demchuk A.M., Dávalos A., Majoie C.B., van Der Lugt A., De Miquel M.A. (2016). Endovascular thrombectomy after large-vessel ischaemic stroke: A meta-analysis of individual patient data from five randomised trials. Lancet.

[7] Powers W.J., Rabinstein A.A., Ackerson T., Adeoye O.M., Bambakidis N.C., Becker K., Biller J., Brown M., Demaerschalk B.M., Hoh B. (2019). Guidelines for the Early Management of Patients With Acute Ischemic Stroke: 2019 Update to the 2018 Guidelines for the Early Management of Acute Ischemic Stroke: A Guideline for Healthcare Professionals From the American Heart Association/American Stroke Association. Stroke.

[8] Turc G., Tsivgoulis G., Audebert H.J., Boogaarts H., Bhogal P., De Marchis G.M., Fonseca A.C., Khatri P., Mazighi M., de La Ossa N.P. (2019). European Stroke Organisation (ESO)—European Society for Minimally Invasive Neurological Therapy (ESMINT) Guidelines on Mechanical Thrombectomy in Acute Ischaemic StrokeEndorsed by Stroke Alliance for Europe (SAFE). Eur. Stroke J..

[9] Vidale S., Agostoni E. (2017). Endovascular Treatment of Ischemic Stroke: An Updated Meta-Analysis of Efficacy and Safety. Vasc. Endovasc. Surg..

[10] Matias-Guiu J.A., Gil A., Serna-Candel C., Simal P., García-García A.M., Egido J.A., Matías-Guiu J., López-Ibor L. (2013). Endovascular treatment of distal internal carotid artery occlusions with retrievable stents. Eur. Neurol..

[11] Xing P.F., Yang P.F., Li Z.F., Zhang L., Shen H.J., Zhang Y.X., Zhang Y.W., Liu J.M. (2020). Comparison of Aspiration versus Stent Retriever Thrombectomy as the Preferred Strategy for Patients with Acute Terminal Internal Carotid Artery Occlusion: A Propensity Score Matching Analysis. AJNR Am. J. Neuroradiol..

[12] Flint A.C., Duckwiler G.R., Budzik R.F., Liebeskind D.S., Smith W.S. (2007). Mechanical thrombectomy of intracranial internal carotid occlusion: Pooled results of the MERCI and Multi MERCI Part I trials. Stroke.

[13] McTaggart R.A., Tung E.L., Yaghi S., Cutting S.M., Hemendinger M., Gale H.I., Baird G.L., Haas R.A., Jayaraman M.V. (2017). Continuous aspiration prior to intracranial vascular embolectomy (CAPTIVE): A technique which improves outcomes. J. Neurointerv. Surg..

[14] Chueh J.Y., Kühn A.L., Puri A.S., Wilson S.D., Wakhloo A.K., Gounis M.J. (2013). Reduction in distal emboli with proximal flow control during mechanical thrombectomy: A quantitative in vitro study. Stroke.

[15] Mokin M., Nagesh S.V., Ionita C.N., Mocco J., Siddiqui A.H. (2016). Stent retriever thrombectomy with the Cover accessory device versus proximal protection with a balloon guide catheter: In vitro stroke model comparison. J. Neurointerv. Surg..

[16] Zaidat O.O., Mueller-Kronast N.H., Hassan A.E., Haussen D.C., Jadhav A.P., Froehler M.T., Jahan R., Ali Aziz-Sultan M., Klucznik R.P., Saver J.L. (2019). Impact of Balloon Guide Catheter Use on Clinical and Angiographic Outcomes in the STRATIS Stroke Thrombectomy Registry. Stroke.

[17] Baek J.H., Kim B.M., Kang D.H., Heo J.H., Nam H.S., Kim Y.D., Hwang Y.H., Kim Y.W., Kim Y.S., Kim D.J. (2019). Balloon Guide Catheter Is Beneficial in Endovascular Treatment Regardless of Mechanical Recanalization Modality. Stroke.

[18] Pederson J.M., Reierson N.L., Hardy N., Touchette J.C., Medam S., Barrett A., Schmidt M., Brinjikji W., Kallmes D.F., Kallmes K.M. (2021). Comparison of Balloon Guide Catheters and Standard Guide Catheters for Acute Ischemic Stroke: A Systematic Review and Meta-Analysis. World Neurosurg..

[19] Brinjikji W., Starke R.M., Murad M.H., Fiorella D., Pereira V.M., Goyal M., Kallmes D.F. (2018). Impact of balloon guide catheter on technical and clinical outcomes: A systematic review and meta-analysis. J. Neurointerv. Surg..

[20] Nguyen T.N., Castonguay A.C., Nogueira R.G., Haussen D.C., English J.D., Satti S.R., Chen J., Farid H., Borders C., Veznedaroglu E. (2019). Effect of balloon guide catheter on clinical outcomes and reperfusion in Trevo thrombectomy. J. Neurointerv. Surg..

[21] Podlasek A., Dhillon P.S., Jewett G., Shahein A., Goyal M., Almekhlafi M. (2021). Clinical and Procedural Outcomes with or without Balloon Guide Catheters during Endovascular Thrombectomy in Acute Ischemic Stroke: A Systematic Review and Meta-analysis with First-line Technique Subgroup Analysis. AJNR Am. J. Neuroradiol..

[22] Kwak J.H., Zhao L., Kim J.K., Park S., Lee D.G., Shim J.H., Lee D.H., Kim J.S., Suh D.C. (2014). The outcome and efficacy of recanalization in patients with acute internal carotid artery occlusion. AJNR Am. J. Neuroradiol..

[23] Liebeskind D.S., Flint A.C., Budzik R.F., Xiang B., Smith W.S., Duckwiler G.R., Nogueira R.G. (2015). Carotid I’s, L’s and T’s: Collaterals shape the outcome of intracranial carotid occlusion in acute ischemic stroke. J. Neurointerv. Surg..

[24] European Stroke Organisation (ESO), Executive Committee, ESO Writing Committee (2008). Guidelines for management of ischaemic stroke and transient ischaemic attack 2008. Cerebrovasc. Dis..

[25] Velasco A., Buerke B., Stracke C.P., Berkemeyer S., Mosimann P.J., Schwindt W., Alcázar P., Cnyrim C., Niederstadt T., Chapot R. (2016). Comparison of a Balloon Guide Catheter and a Non-Balloon Guide Catheter for Mechanical Thrombectomy. Radiology.

[26] Romano D.G., Frauenfelder G., Diana F., Saponiero R. (2022). JET 7 catheter for direct aspiration in carotid T occlusions: Preliminary experience and literature review. Radiol. Med..

[27] Maus V., Hüsken S., Kalousek V., Karwacki G.M., Nordmeyer H., Kleffner I., Weber W., Fischer S. (2021). Mechanical Thrombectomy in Acute Terminal Internal Carotid Artery Occlusions Using a Large Manually Expandable Stentretriever (Tiger XL Device): Multicenter Initial Experience. J. Clin. Med..

[28] Pero G., Dória H.M., Piano M., Macera A., Quilici L., Cervo A. (2023). Intracranial Carotid Occlusions: ADAPT versus SAVE and the role of Balloon Guide Catheters. Clin. Neuroradiol..

[29] Rubiera M., Ribo M., Delgado-Mederos R., Santamarina E., Delgado P., Montaner J., Alvarez-Sabín J., Molina C.A. (2006). Tandem internal carotid artery/middle cerebral artery occlusion: An independent predictor of poor outcome after systemic thrombolysis. Stroke.

